# Prevalence, demographics and clinical characteristics of Internet addiction among Chinese adolescents with schizophrenia

**DOI:** 10.3389/fpsyt.2024.1398479

**Published:** 2024-05-03

**Authors:** Yunhui Zhong, Yibo Li, Anquan Hu, Xiang-Yang Zhang

**Affiliations:** ^1^ The Third People’s Hospital of Ganzhou, Ganzhou, China; ^2^ Department of Psychiatry and Psychology, College of Basic Medical Sciences, Tianjin Medical University, Tianjin, China; ^3^ Chinese Academy of Sciences (CAS) Key Laboratory of Mental Health, Institute of Psychology, Chinese Academy of Sciences, Beijing, China; ^4^ Department of Psychology, University of Chinese Academy of Sciences, Beijing, China

**Keywords:** adolescents, Chinese, internet addiction, schizophrenia, prevalence

## Abstract

**Introduction:**

Several studies have reported that Internet addiction (IA) is more prevalent in the psychiatric disorder population. However, the prevalence of IA and its relationship with clinical variables among Chinese adolescents with schizophrenia is unclear. This study sought to investigate the prevalence of IA and its clinical correlates in Chinese adolescents with schizophrenia, which has not yet been reported.

**Methods:**

Seven hundred and six inpatient adolescents with schizophrenia were recruited. All patients underwent Young’s Internet Addiction Test (IAT) to measure Internet addiction, as well as the Positive and Negative Syndrome Scale (PANSS) for psychopathology.

**Results:**

Our results showed that 186 patients had a total IAT score of 50 or more, bringing the prevalence of IA to 26.3%. Girls (21.7%, 92/424) were less likely to have combined IA than boys (33.3%, 94/282). Compared to those patients without IA, patients with IA had better socioeconomic status, higher probability of living in the city, higher levels of depressive symptoms, excited symptoms, and lower levels of concrete symptoms and PANSS total scores (all p<0.05). Further binary logistic regression analysis indicated that good socioeconomic status, living in the city and PANSS total scores were significantly associated with IA. In addition, correlation analysis showed significant correlations between IA total score and the following parameters: good socioeconomic status, living in the city, negative subscore, concrete subscore, depression subscore, excited subscore and PANSS total score (Bonferroni corrected all p <0.05).

**Conclusion:**

The results of this study indicate that the prevalence of IA in Chinese adolescents with schizophrenia is higher than that in the general population. Several demographic and clinical variables are risk factors for IA in adolescents with schizophrenia.

## 1 Introduction

Nowadays, the Internet has become an integral part of daily life, bringing great convenience to people in communicating with others, obtaining relaxing entertainment available, and searching for information. The use of the Internet is becoming more and more common in daily life, especially amongst the youth population. However, the Internet has also had negative impacts on individual, which has been described as Internet addiction (IA) or problematic Internet use (1, 2). According to Kandell (3), it is well established that IA is defined as a psychological dependence on the Internet characterized by an increasing investment of resources in Internet-related activities, unpleasant feelings when offline, increasing tolerance for the effects of being online, and denial of problematic behaviors. Although IA has not been diagnosed as a clinical disorder, the Diagnostic and Statistical Manual of Mental Disorders, Fifth Edition, has included Internet Gaming Disorder as an emerging problem for further research (4). There is growing evidence of an association between IA and a range of negative outcomes in adolescents, such as interpersonal problems (5), insomnia (6), suicidal ideation (7, 8), smoking problem behaviors (9), and mental health (10–12). IA is a prominent public health problem among adolescents that has attracted worldwide attention (13). A meta-analysis of data from 31 nationwide studies in seven regions of the world found an overall prevalence of IA of 6% (14). Specifically, the prevalence rates of IA in China range from 6% to 17.4% (15–17).

Several studies have reported that IA is more prevalent among people with psychiatric disorder (18, 19). For example, the prevalence of IA and use of portable games among adolescents with autism spectrum disorder is substantially higher than in the general population (10, 20). Dieris-Hirche, Bottel (21) reported that depressed patients exhibited a high prevalence of co-occurrence of maladaptive Internet use and IA, suggesting that the co-occurrence of depression and IA should be noted and considered in psychiatric service. In addition, Ko, Yen (22) also determined that adolescents with problematic alcohol use were more likely to have IA. However, only a few studies have investigated the prevalence of IA in patients with schizophrenia. One recent study by Lee, Chung (23) examined the contribution of stress and coping strategies to problematic internet use in Korean adults with schizophrenia spectrum disorders. They reported that patients with schizophrenia spectrum disorders showed a high prevalence of problematic Internet use at 22.0%. However, it is noteworthy that no studies have reported the prevalence of IA among adolescents with schizophrenia.

To our knowledge, there are no studies on schizophrenia with Internet addiction in the Chinese Han population, especially in the adolescent population. Therefore, the main objectives of this were to 1) investigate the prevalence of IA among Chinese adolescents with schizophrenia, 2) compare the prevalence in this study sample with previous surveys conducted in the general adolescent population in China, and 3) determine the demographic and clinical characteristics of IA among Chinese adolescents with schizophrenia. We hypothesized that the prevalence of IA in Chinese adolescents with schizophrenia would be much higher than in the general adolescent population and that some clinical variables would be risk factors for IA.

## 2 Methods

### 2.1 Setting and subjects

The study was conducted at the Third People’s Hospital of Ganzhou city from June, 2018 to October, 2021. This hospital is a public psychiatric hospital owned by Ganzhou city, with a total population of approximately 9 million. We approached all inpatients using a cross-sectional naturalistic design.

We recruited 706 patients who met the following criteria: 1) 13-18 years old and Han Chinese; 2) diagnosed with schizophrenia or schizoaffective disorder by two psychiatrists using the Chinese version of Structured Clinical Interview for DSM-IV (SCID); and 3) no other psychiatric diagnoses.

Participants were excluded when the following criteria were met: 1) having severe physical disorders; 2) having drug and alcohol abuse/dependence except for nicotine; 3) being unable to provide written formal informed consent.

The study was approved by the Institutional Review Board (IRB) of the Third People’s Hospital of Ganzhou, and written informed consent was obtained from all patients or their legal guardians after receiving a full explanations regarding the study procedures.

The sample size was determined using the formula n = Z^2^p(1-p)/d^2^. n = sample size; Z= 95% confidence interval, equal to 1.96; p = expected prevalence, equal to 0.22 based on a study of adult schizophrenia (23); d = 0.05 (5%), marginal error. The final sample size included in this study (n = 706) was significantly larger than the required sample size (n = 264), which indicates that our sample size had adequate power.

### 2.2 Data collection and measurements

A detailed questionnaire was completed collecting general information, socio-demographic characteristics, and medical and psychological conditions. Also, the researchers collected available records and collateral data sources (from family and/or treating clinician). The following data were collected for each patients: sex, age, education, family structure, socioeconomic status, place of residence, age of onset, age at first hospitalization, number of hospitalizations, and duration of illness. Gender- and age-specific body mass index (BMI) cutoff points recommended by the Working Group for Obesity in China (WGOC) were used to define overweight and obesity (24).

Patients’ psychopathology was assessed using the Positive and Negative Syndrome Scale (PANSS) (25) by four psychiatrists who were blind to the clinical status of patients. To ensure consistency and reliability of the measurement throughout the study, these four psychiatrists, who had at least 5 years of clinical experience, simultaneously attended a training course in the use of the PANSS before the study began. After training, their inter-rater correlation coefficients for the PANSS total score were maintained at 0.83. In addition, the five-factor model of the PANSS proposed by Wallwork, Fortgang (26) was used to assess the psychopathology of patients. The five factors include a positive factor (items P1, P3, P5, G9), a negative factor (items N1, N2, N3, N4, N6, G7), a concrete/disorganized factor (items P2, N5, G11), an excited factor (items P4, P7, G8, G14) and a depressed factor (items G2,G3 and G6).

IA was assessed by a Chinese version of the Internet addiction test (IAT) (27, 28), which consisted of 20 items rated on a five-point Likert scale, covering the extent to which their Internet use affects their daily life, social life, productivity, sleeping pattern, and feelings. The total score ranges from 20 to 100, with higher score indicating more serious problems caused by internet use. IA was assessed by summing the IAT scores with a cut-off point of ≥ 50 being classified as IA (29).

### 2.3 Data analysis

In the present study, the Kolmogorov-Smirnov one-sample test was used to measure the normal distribution of continuous data. Since all continuous data conformed to a normal distribution, comparisons of demographic and clinical variables between the IA and non-IA groups were performed using independent samples *t*-tests for continuous variables and chi-square tests for categorical variables. Prevalence of IA was described as a percentage and analyzed using chi-square tests. Bonferroni correction was used to adjust for multiple testing. A binary logistic regression model was used to determine which factors had a significant effect on IA. In addition, odds ratios (OR) for categorical variables were calculated by X^2^ tests and adjusted ORs were calculated by binary logistic methods after controlling for confounders.

All statistical analyses were performed in SPSS (version 21.0; SPSS Inc., Chicago, Illinois, USA). All p-values were 2-tailed, and the significance level was set at 0.05.

## 3 Results

### 3.1 Demographic and clinical characteristics of Chinese adolescents with schizophrenia

A total of 706 inpatients, including 282 boys and 424 girls, were included in our study. The mean age of the patients was 15.41 [standard deviation (SD) =1.45], ranging from 13 to 18 years. The mean duration of education was 8.48 years (SD =1.38) with a range of 6 to 12 years. The mean age of onset was 13.84 years (SD =2.14), with a range of 6 to 18 years. The mean duration of illness was 20.81 months (SD =23.12), with a range of 1 to 240 months. The average of age of hospitalization at onset was 14.45 years (SD =1.69), ranging from 9 to 18 years. The mean number of hospitalizations was 3.10 (SD =2.19) with a range of 1 to 17.

The mean PANSS scores were: positive, 14.82 ± 6.83; negative, 23.93 ± 8.40; concrete, 5.06 ± 1.65; depressive, 8.94 ± 4.20; excited, 13.39 ± 7.62 and total score, 98.06 ± 15.88.

### 3.2 Prevalence, demographic and clinical variables in IA versus non-IA participants

The prevalence of IA was 26.3% (186/706). Girls (21.69%, 92 of 424) were less likely than boys (33.33%, 94 of 282) to have a comorbid IA (X^2^ = 11.82, df=1, p=0.001). After controlling for age and education, IA rates for boys were 1.80 times higher than for girls (B=0.59, Wald statistic=11.674, p <0.001, OR=1.804, 95%CI=1.286-2.531).

As shown in **Table 1**, the clinical and sociodemographic characteristics of IA and non-IA adolescents with schizophrenia were compared. IA patients were more likely to be boys (p=0.001), have better socioeconomic status (p=0.000), live in urban areas (p=0.000), higher levels of depressive symptoms (p=0.000), and excited symptoms (p=0.000), but lower levels of concrete symptoms (p=0.043) and PANSS total score (p=0.000).

**Table 1:** Demographic and clinical characteristics between the IA and non-IA in adolescents with schizophrenia (n = 706).

Characteristics	Category	Total	IA	Non-IA	t/X^2^	*P*-value
			n (%)	n (%)		
**Gender**	Boys	282 (39.9%)	94 (50.5%)	188 (36.2%)	11.816	0.001
	Girls	424 (60.1%)	92 (49.5%)	332 (63.8%)		
**Family structure**	Living with parents	668 (94.6%)	180 (96.8%)	488 (93.8%)	2.306	0.129
	Single parent family	38 (5.4%)	6 (3.2%)	32 (6.2%)		
**Being the only child**	Yes	150 (21.2%)	46 (24.7%)	104 (20.0%)	1.833	0.176
	No	556 (78.8%)	140 (75.3%)	416 (80.0%)		
**Family history**	Yes	114 (16.1%)	30 (16.1%)	84 (16.2%)	0.000	0.994
	No	592 (83.9%)	156 (83.9%)	426 (83.8%)		
**Socioeconomic status**	Good	512 (72.5%)	170 (91.4%)	342 (65.8%)	45.154	0.000
	Bad	194 (27.5%)	16 (8.6%)	178 (34.2%)		
**Residence**	Urban	178 (25.2%)	82 (44.1%)	96 (18.5%)	47.706	0.000
	Rural	528 (74.8%)	104 (55.9%)	424 (81.5%)		
**Weight**	Normal weight	468 (66.3%)	112 (60.2%)	356 (68.5%)	6.012	0.057
	Overweight	162 (22.9%)	46 (24.7%)	116 (22.3%)		
	Obesity	76 (10.8%)	28 (15.1%)	48 (9.2%)		
Age (years)	mean ± SD	15.411 ± 1.455	15.366 ± 1.413	15.427 ± 1.473	0.282	0.622
Education (years)	mean ± SD	8.484 ± 1.381	8.398 ± 1.328	8.515 ± 1.402	0.012	0.319
Age at onset (years)	mean ± SD	13.839 ± 2.141	13.743 ± 2.553	18.873 ± 1.978	9.843	0.474
Duration of illness (months)	mean ± SD	20.810 ± 23.141	21.215 ± 24.707	20.665 ± 22.603	12.067	0.781
Age of hospitalization at onset (years)	mean ± SD	14.450 ± 1.688	14.387 ± 1.782	14.473 ± 1.657	1.439	0.551
Hospitalization numbers	mean ± SD	3.099 ± 2.187	3.258 ± 2.582	3.042 ± 2.054	0.076	0.248
Positive subscore	mean ± SD	14.824 ± 6.833	15.839 ± 7.174	14.462 ± 6.684	1.306	0.063
Negative subscore	mean ± SD	23.932 ± 8.406	22.591 ± 8.441	24.412 ± 8.358	0.053	0.071
Concrete subscore	mean ± SD	5.060 ± 1.648	4.849 ± 1.799	5.135 ± 1.587	0.600	0.043
Depressive subscore	mean ± SD	8.943 ± 4.203	10.86 ± 4.636	8.258 ± 3.819	2.542	0.000
Excited subscore	mean ± SD	13.391 ± 7.628	18.602 ± 6.498	11.527 ± 7.131	7.805	0.000
PANSS Total	mean ± SD	98.068 ± 15.880	96.15 ± 14.11	103.43 ± 19.102	31.763	0.000

Values expressed as no. (%) or mean (± standard deviation). IA, internet addiction; Non-IA, without internet addiction.

### 3.3 Correlation of IA with demographic and clinical characteristics

The mean total IA score for all patients was 41.90 ± 18.93. Pearson correlation analysis showed that IA total score was associated with gender (r=0.201, p<0.01), socioeconomic status (r=0.271, p<0.01), place of residence (r=0.262, p<0.01), negative score (r=-0.123, p<0.01), concrete score (r=-0.151, p<0.01), depression score (r=0.328, p<0.01), excited score (r=0.454, p<0.01), and total PANSS score (r=0.184, p<0.01). All these associations remained significant after Bonferroni correction (all p<0.05). **Table 2** shows the correlations between IA and demographic data or clinical characteristics.

**Table 2:** Association between IA and demographic data and clinical characteristics.

characteristics	1	2	3	4	5	6	7	8	9	10	11	12
1.IA	1											
2.Age	0.011	1										
3.Gender	0.201**	-0.100**	1									
4.Socioeconomic status	0.271**	-0.135**	0.178**	1								
5.Residence	0.262**	0.007	0.153**	0.299**	1							
6.Weight	0.041	0.055	0.049	-0.034	-0.038	1						
7.Positive subscore	0.061	-0.090*	0.127**	-0.184**	-0.001	0.009	1					
8.Negative subscore	-0.123**	-0.188**	0.155**	0.424**	0.090*	-0.030	-0.333**	1				
9.Concrete subscore	-0.151**	-0.178**	0.086*	0.178**	0.104**	0.032	0.069	0.229**	1			
10.Depressive subscore	0.328**	0.126**	-0.243**	-0.548**	-0.368**	-0.021	0.226**	-0.255**	-0.127**	1		
11.Excited subscore	0.454**	-0.01	-0.317**	-0.428**	-0.428**	0.021	0.220**	-0.270**	0.002	0.451**	1	
12.PANSS Total	0.184**	-0.186**	0.019	-0.094*	-0.143**	-0.013	0.460**	0.356**	0.353**	0.403**	0.537**	1

*P<0.05, **P<0.01.

We then focused on risk factors for IA in adolescents with schizophrenia. Variables with significant differences in univariate analysis were included in logistic regression analysis (Backward: Wald). As shown in **Table 3**, the risk factors for IA were as follows: socioeconomic status(good) (B=0.83, P<0.01, OR=2.293), place of residence(urban) (B=0.658, P<0.01, OR=1.93), and PANSS total score (B=-0.24, P<0.01, OR=0.976).

**Table 3:** Binary logistic regression analysis showing the risk factors associated with IA.

Variables	B	Wald X^2^(df=1)	*P*-value	OR	95% CI
Gender, boys	-0.21	0.012	0.914	0.979	0.663-1.444
Socioeconomic status, good	0.830	7.504	0.006	2.293	1.266-4.153
Residence, urban	0.658	10.061	0.002	1.930	1.286-2.898
Concrete score	0.103	3.172	0.075	1.108	0.990-1.241
Excited score	1.61	63.138	0.091	0.892	0.867-0.917
PANSS total score	-0.24	8.734	0.003	0.976	0.96-0.992

CI, confidence interval; IA, internet addiction; OR, odds ratio.

## 4 Discussion

To our best knowledge, this was the first study to examine the prevalence and clinical correlates of IA among inpatients with schizophrenia in a Chinese adolescent population. We found that 26.3% of adolescents with schizophrenia met the criteria for IA and that significant clinical correlates of IA in this population were having good socioeconomic status, living in an urban area, and having a low PANSS total score.

Based on the IAT, Lam, Peng (30) found that 10.8% of adolescents aged 13-18 year in China were moderately to severely addicted to the Internet. Cao and Su (31) reported that 18.2% of junior high school students in China were classified as IA based on the same test. Notably, the prevalence of IA among adolescents with schizophrenia in our study was 26.3%, indicating a relatively high prevalence of IA compared to the prevalence in the general adolescent population in China. Schizophrenia is a well-known risk factor for addictive disorders (32). Brunette, Mueser (33) showed that more than half of the patients with first-episode psychosis had a substance use disorders. Also, Desai and Potenza (34) found that individuals with schizophrenia/schizoaffective disorder may be at particularly high risk for problem and pathological gambling. Taken together, these findings suggest that higher prevalence of IA may occur in patients with schizophrenia, especially in adolescents with schizophrenia. One possible explanation is that dopamine dysregulation has been implicated in both schizophrenia and addiction disorders (35, 36). Adolescents with schizophrenia may exhibit dysregulated dopamine transmission, leading to a heightened sensitivity to rewarding stimuli, including those encountered online. This hyperdopaminergic state could contribute to the reinforcing effects of internet use, promoting addictive behaviors.

Another possible explanation for the high prevalence of IA in adolescents with schizophrenia is the perceived stigma of the patients. Perceived and experienced stigma as well as self-stigma are phenomena involving a high proportion of patients with schizophrenia spectrum disorders (37). A previous study by Li, Guo (38) reported that stigma is associated with social withdrawal in patients with schizophrenia, which leads them to lose their jobs or drop out of school. Due to anonymity, people with schizophrenia who experience stigma avoid face-to-face contact whenever possible, preferring to use the Internet to exchange information (39, 40). In this way, it is relatively easy to become addicted to the Internet in the absence of control.

We also found a significant gender difference in the prevalence of comorbid IA among adolescents with schizophrenia, showing a higher prevalence among males than females (33.3% vs. 21.7%). Numerous studies have shown gender differences in the prevalence of IA among the general adolescent population in China (41, 42). According to Chen, Kang (41), this may be due to the fact that males are more involved in online activities such as gaming, pornography, and gambling, which may lead to pathological Internet use. Consistent with the results for the general adolescent population, our results also suggest that the prevalence of IA is higher among males than females. The gender differences in IA found in this study may be due to the fact that females with schizophrenia have less social cognitive impairment than males (43).

Another finding of our present study was that good socioeconomic status and living in an urban area were independently associated with IA. Our study showed that Internet use among adolescents with schizophrenia was influenced by environmental factors. Good socioeconomic status and living in an urban area gave patients more access to the Internet. Previous findings reported that more Internet use was associated with higher economic income (44, 45) and living in urban areas (46).

Consistent with previous studies (2, 47), we also found that IA was associated with patients psychopathology. In our current study, those patients with lower PANSS levels were significantly associated with an increased risk of IA. One reason for this may be that well-conditioned patients can successfully use the Internet. Higher PANSS scores mean that patients are in worse condition, such attention deficit or delusional interpretations, as well as motivational deficits, which lead to rejection of Internet use (48).

Several limitations of this study should be noted. First, the sample was drawn from a general hospital in Ganzhou, mainland China. Therefore, the results are not necessarily representative of the entire mainland China, let alone other Asian countries. Second, regarding our sample of study subjects, the participants in this study were recruited from an acute inpatient psychiatry unit. Therefore, the results of the study cannot be generalized to outpatients or community patients. Third, our investigation was a cross-sectional design, which precludes proof of causality. Fourth, we did not collect data on all the variables that could be related to IA, such as the purpose of Internet use and medical conditions, including medications. Fifth, previous studies have shown that, there is a positive relationship between internet addiction and obesity (49, 50). However, due to the sample size of the subjects, the correlation between internet addiction and obesity in this study did not reach a significant level, which requires further research in the future.

In summary, our results show the prevalence of comorbid IA among Chinese adolescents with schizophrenia was 26.3%, suggesting that IA is common in this population. Furthermore, patients with IA have good socioeconomic status, live in urban areas, and have low PANSS score. The present study may suggest that IA in adolescents with schizophrenia is influenced by environmental and psychopathology factors. Therefore, clinicians working with adolescents with schizophrenia should consider IA and related factors and intervene with IA.

## Data availability statement

The raw data supporting the conclusions of this article will be made available by the authors, without undue reservation.

## Ethics statement

The studies involving humans were approved by The Institutional Review Board (IRB) of the Third People’s Hospital of Ganzhou. The studies were conducted in accordance with the local legislation and institutional requirements. Written informed consent for participation in this study was provided by the participants’ legal guardians/next of kin.

## Author contributions

YZ: Investigation, Writing – original draft. YL: Methodology, Writing – review & editing. AH: Data curation, Investigation, Writing – original draft. X-YZ: Conceptualization, Methodology, Writing – review & editing.
